# Impairment of Coronary Endothelial Function by Hypoxia-Reoxygenation Involves TRPC3 Inhibition-mediated K_Ca_ Channel Dysfunction: Implication in Ischemia-Reperfusion Injury

**DOI:** 10.1038/s41598-017-06247-3

**Published:** 2017-07-19

**Authors:** Xiang-Chong Wang, Wen-Tao Sun, Jie Fu, Jun-Hao Huang, Cheuk-Man Yu, Malcolm John Underwood, Guo-Wei He, Qin Yang

**Affiliations:** 1 Division of Cardiology, Department of Medicine and Therapeutics, Institute of Vascular Medicine, Li Ka Shing Institute of Health Sciences, Institute of Innovative Medicine, The Chinese University of Hong Kong, Hong Kong, China; 2 Division of Cardiothoracic Surgery, Department of Surgery, The Chinese University of Hong Kong, Hong Kong, China; 3 GuangZhou Sport University, GuangZhou, China; 4 TEDA International Cardiovascular Hospital, Chinese Academy of Medical Sciences, Tianjin, China

## Abstract

Despite increasing knowledge of the significance of calcium-activated potassium (K_Ca_) and canonical transient receptor potential (TRPC) channels in endothelial physiology, no studies so far have investigated the link between these two distinct types of channels in the control of vascular tone in pathological conditions. We previously demonstrated that hypoxia-reoxygenation (H-R) inhibits endothelial K_Ca_ and TRPC3 channels in porcine coronary arteries (PCAs). The present study further investigated whether modulation of TRPC3 is involved in H-R-induced K_Ca_ channel inhibition and associated vasodilatory dysfunction using approaches of wire myography, whole-cell voltage-clamp, and coimmunoprecipitation. Pharmacological inhibition or siRNA silencing of TRPC3 significantly suppressed bradykinin-induced intermediate- and small-conductance K_Ca_ (IK_Ca_ and SK_Ca_) currents in endothelial cells of PCAs (PCAECs). TRPC3 protein exists in physical association with neither IK_Ca_ nor SK_Ca_. In H-R-exposed PCAECs, the response of IK_Ca_ and SK_Ca_ to bradykinin-stimulation and to TRPC3-inhibition was markedly weakened. Activation of TRPC3 channels restored H-R-suppressed K_Ca_ currents in association with an improved endothelium-derived hyperpolarizing factor (EDHF)-type vasorelaxation. We conclude that inhibition of TRPC3 channels contributes to H-R-induced suppression of K_Ca_ channel activity, which serves as a mechanism underlying coronary endothelial dysfunction in ischemia-reperfusion (I-R) injury and renders TRPC3 a potential target for endothelial protection in I-R conditions.

## Introduction

The role of intermediate and small conductance calcium-activated K^+^ (IK_Ca_ and SK_Ca_) channels of endothelial cells in the control of vascular tone has been well documented. Opening of IK_Ca_ and SK_Ca_ channels in response to increased cytosolic Ca^2+^ is the mechanism underlying the classical endothelium-derived hyperpolarizing factor (EDHF) pathway^1^. In addition, activation of these channels is also involved in the regulation of eNOS activity and NO function^2, 3^. Disturbed function of IK_Ca/_SK_Ca_ channels has been reported to be associated with endothelial dysfunction in cardiovascular pathologies, e.g., hypertension and diabetes^4^.

Members of the transient receptor potential (TRP) protein superfamily form nonselective cation channels that are of great importance in [Ca^2+^]_i_ regulation in vascular endothelial cells^5, 6^. It was reported that native canonical TRP3 (TRPC3) channels contribute to constitutive and ATP-dependent Ca^2+^ influx in human coronary artery endothelial cells^7^ and TRPC3-mediated Ca^2+^ influx was demonstrated to be essential for NO production and endothelium-dependent relaxation in porcine coronary and human arteries^8, 9^. Altered expression and/or activity of TRPC3 have been associated, directly or indirectly, with endothelial dysfunction and endothelium-related vascular disease^10^.

Ischemia-reperfusion (I-R) injury constitutes one of the major causes of cardiovascular morbidity and mortality^11^. Vascular endothelium is vulnerable to I-R injury^12, 13^. Previous research provided evidence in support of the role of K_Ca_ channel dysregulation in I-R-induced endothelial dysfunction^14, 15^. In our *in vitro* studies of I-R in porcine coronary arteries, we demonstrated that hypoxia-reoxygenation (H-R) suppresses IK_Ca_ and SK_Ca_ channel currents in endothelial cells and such inhibition is responsible for the compromised EDHF-mediated vasorelaxation and smooth muscle hyperpolarization^16^. In addition, through inhibiting the expression of TRPC3 protein on endothelial cell membrane, H-R significantly reduces TRPC3 channel activity and Ca^2+^ influx via TRPC3, leading to decreased NO production and vasorelaxation^8^.

Although knowledge of the significance of IK_Ca/_SK_Ca_ and TRPC3 channels in endothelial pathophysiology has been growing, very few studies to date have attempted to explore the link between these two distinct types of channels in the control of vascular tone. With using pressure myograph and sharp electrode intracellular recording, Senadheera and colleagues first reported that TRPC3 facilitates IK_Ca/_SK_Ca_-dependent endothelial cell hyperpolarization and vasodilation in rat mesenteric arteries^17^. TRPC3-mediated regulation of SK_Ca_ channel activation was further revealed in mouse cerebral circulation^18^. In agreement with these findings, our study in porcine coronary arteries suggested an important role of TRPC3 channels in mediating EDHF-type relaxation^19^. Despite these pieces of evidence linking TRPC3 and K_Ca_ channels in endothelium physiology, whether the functional link between TRPC3 and K_Ca_ involves physical associations is yet to be examined in coronary vasculature. Furthermore, no studies so far have investigated the relationship of these channels in pathological conditions. Whether modulation of TRPC3 channels is involved in IK_Ca_ and SK_Ca_ channel dysfunction related to endothelial I-R injury remains unexplored. In the present study, by using multiple approaches, including whole-cell voltage-clamp recording of channel currents, myograph studies of vasorelaxation, and molecular biology methods, we aimed to provide answers to the above questions.

## Results

### Blockade and knockdown of TRPC3 channels inhibit endothelial IK_Ca_ and SK_Ca_ channel activity

**Figure 1:**
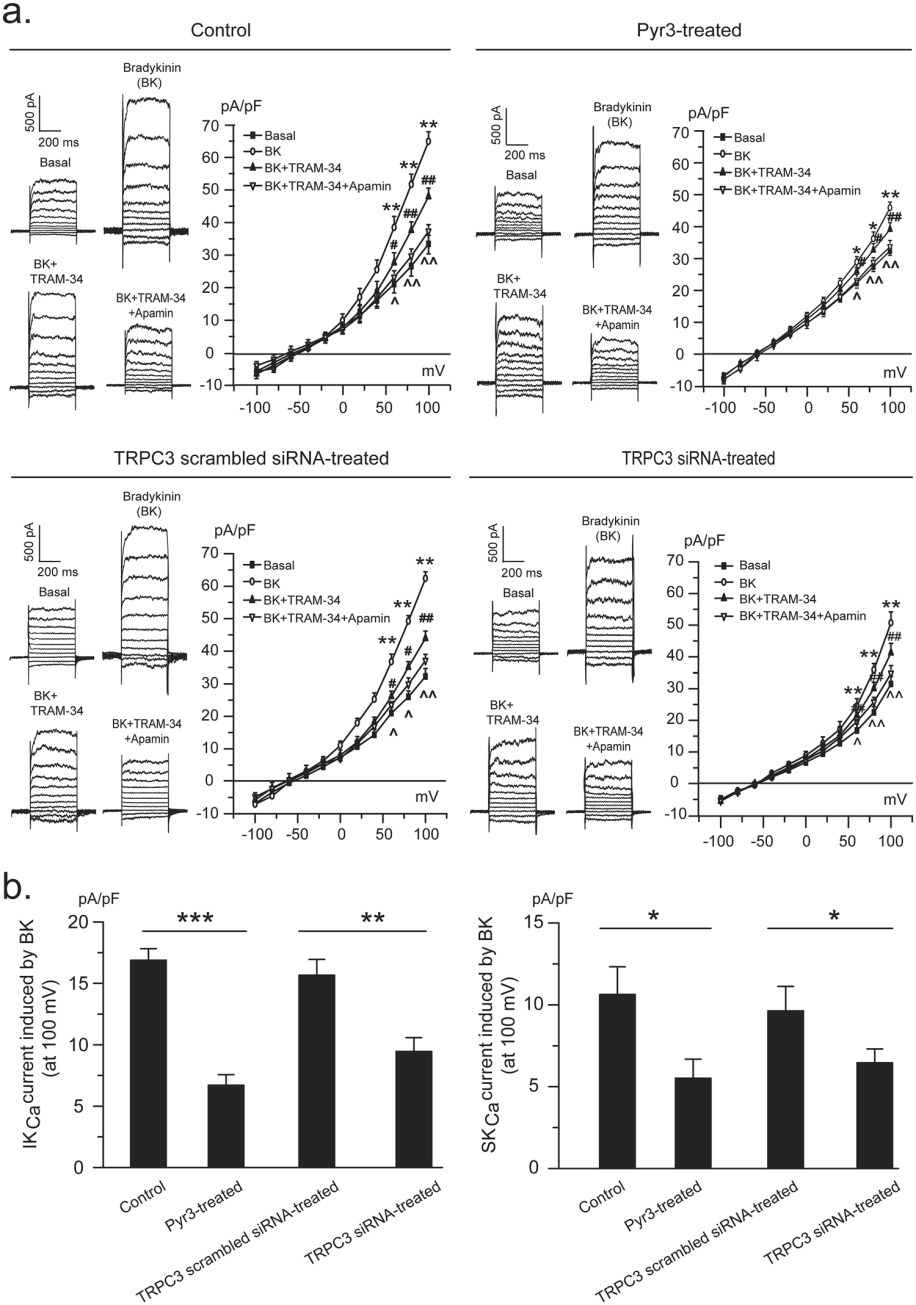
In PCAECs pre-treated with the TRPC3 selective blocker Pyr3 or transfected with TRPC3 specific siRNA, both IK_Ca_ and SK_Ca_ currents in response to bradykinin (BK) were significantly inhibited. (**a**) Representative traces and current-voltage relationship of whole-cell K^+^ current in PCAECs from different treatment groups before and after BK stimulation with further application of the IK_Ca_ channel blocker TRAM-34 and the SK_Ca_ channel blocker apamin. *p < 0.05, **p < 0.01, BK vs. basal; ^#^p < 0.05, ^##^p < 0.01, BK + TRAM-34 vs. BK; ^^^p < 0.05, ^^^^p < 0.01, BK + TRAM-34 + Apamin vs. BK + TRAM-34. (**b**) Summarized data of BK-induced IK_Ca_ and SK_Ca_ currents from 5 independent experiments, each obtained from cell isolates of different heart. *p < 0.05, **p < 0.01, ***p < 0.001, one-way ANOVA and Scheffe post-hoc test.

We examined the role of TRPC3 channels in endothelial K_Ca_ channel activation with using pharmacological tools and siRNA knockdown technique. Pretreatment of PCAECs for 30 min with the specific TRPC3 channel blocker Pyr3 significantly suppressed bradykinin-induced whole-cell K^+^ currents (45.69 ± 2.77 vs. 65.45 ± 3.01 pA/pF, p < 0.01) with the IK_Ca_ channel current decreasing from 16.89 ± 0.93 pA/pF to 6.71 ± 0.85 pA/pF (p < 0.001) and the SK_Ca_ channel current from 10.63 ± 1.69 pA/pF to 5.51 ± 1.17 pA/pF (p < 0.05) (Fig. 1a and b). Further studies using gene silencing approach showed that in PCAECs transfected with TRPC3 specific siRNA, bradykinin-induced K^+^ currents decreased from 62.39 ± 2.06 pA/pF to 50.71 ± 3.49 pA/pF (p < 0.01) and both IK_Ca_ (9.46 ± 1.12 vs. 15.67 ± 1.29 pA/pF, p < 0.01) and SK_Ca_ (6.46 ± 0.84 vs. 9.63 ± 1.49 pA/pF, p < 0.05) currents in response to bradykinin were significantly inhibited (Fig. 1a and b). Successful silencing of the TRPC3 gene was confirmed by the dramatic reduction of channel expression (Supplementary Fig. S1a,b) and the significant inhibition of channel activity which is evidenced by the suppressed response to OAG stimulation (p < 0.05). In response to OAG, membrane current increased from 18.22 ± 2.25 to 23.96 ± 1.01 pA/pF (p < 0.05) in TRPC3 siRNA-transfected cells while increased to 31.04 ± 1.43 pA/pF (p < 0.01) in control cells from 19.19 ± 1.84 pA/pF (Supplementary Fig. S1c). With a subsequent application of Pyr3, the TRPC3 channel current was able to be differentiated and the data further confirmed the loss of TRPC3 channel activity in the gene knockdown cells (4.69 ± 1.08 vs. 8.50 ± 0.35 pA/pF in control, p < 0.05) (Supplementary Fig. S1c,d).

### TRPC3 channels do not physically associate with K_Ca_ channels in PCAECs

**Figure 2:**
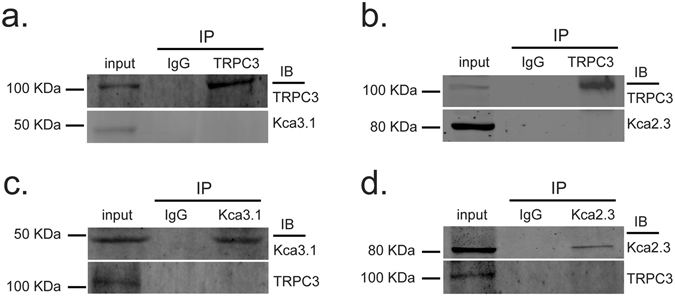
TRPC3 channels do not physically interact with IK_Ca_ and SK_Ca_ channels in PCAECs. The cell lysates were incubated with anti-TRPC3 antibody, and the immunoprecipitates were subjected to immunoblot by anti-K_Ca_3.1 and anti-K_Ca_2.3 antibodies respectively (**a** and **b**). The immunoprecipitation and immunoblot experiments were further performed in reversed order (**c** and **d**). Lysate supernatant (20 μg of protein) was used as input control. Mouse IgG was used as a negative control. Full-length western blot images are presented in Supplementary Figure S2. Experiments in each order were performed for four times with using cell isolates from different hearts.

The results of co-immunoprecipitation experiments showed that in PCAECs, TRPC3 protein co-precipitates with neither K_Ca_3.1 nor K_Ca_2.3 protein, suggesting that TRPC3 channels do not physically associate with K_Ca_ channels in PCAECs (Fig. 2).

### TRPC3 inhibition contributes to the inhibition of endothelial K_Ca_ activity under H-R condition

**Figure 3:**
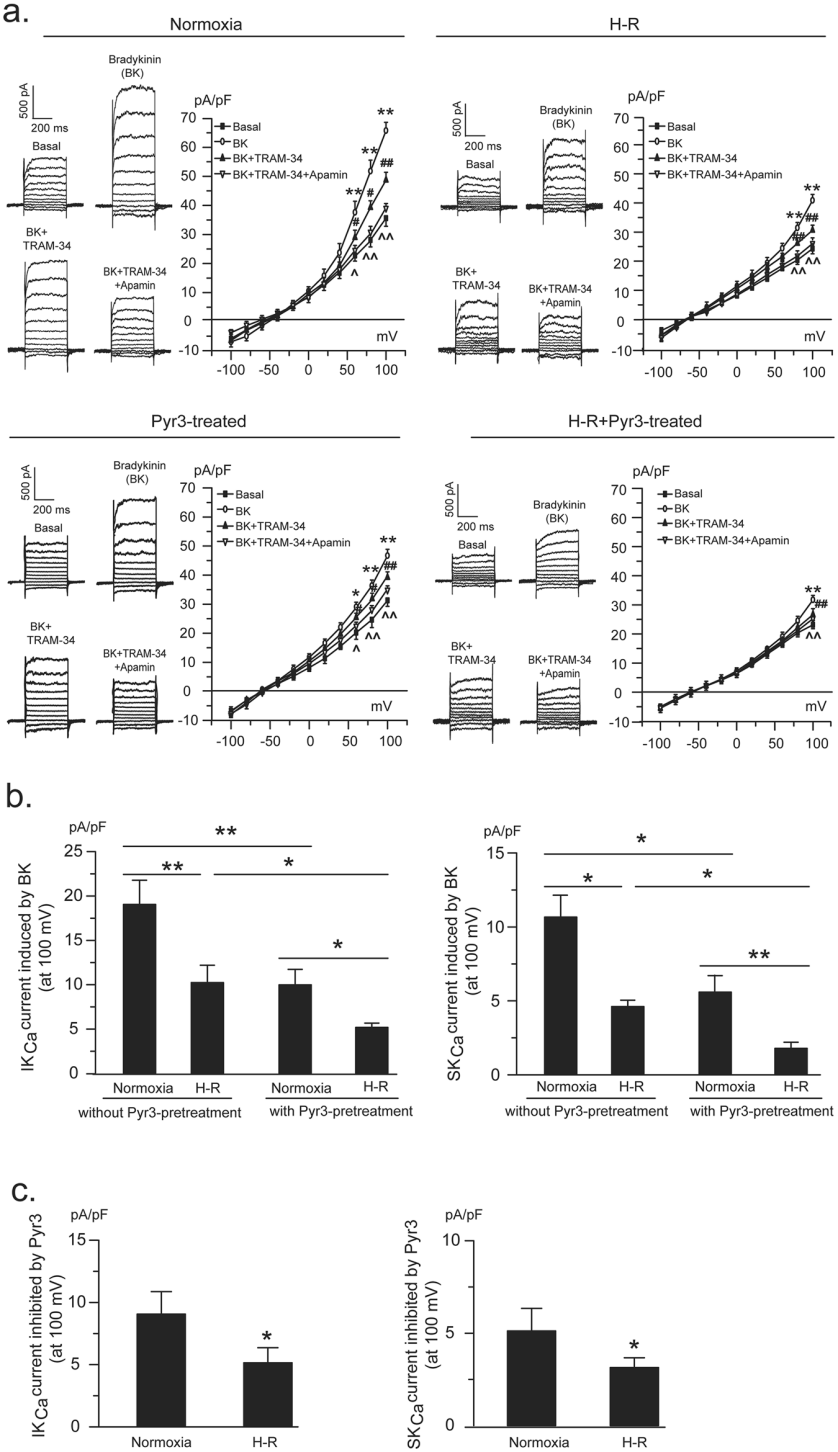
Inhibition of TRPC3 channels contributes to H-R-induced suppression of IK_Ca_ and SK_Ca_ channel activity in PCAECs. Representative traces and current-voltage relationship of whole-cell K^+^ current in PCAECs from different treatment groups before and after bradykinin (BK) stimulation with further application of the IK_Ca_ channel blocker TRAM-34 and the SK_Ca_ blocker apamin (**a**). In H-R group, cells were exposed to 60-min hypoxia and 30-min reoxygenation before patch-clamp recording. *p < 0.05, **p < 0.01, BK vs. basal; ^#^p < 0.05, ^##^p < 0.01, BK + TRAM-34 vs. BK; ^^^p < 0.05, ^^^^p < 0.01, BK + TRAM-34 + Apamin vs. BK + TRAM-34. Pre-treatment of PCAECs with the selective TRPC3 channel blocker Pyr3 significantly inhibited BK-induced IK_Ca_ and SK_Ca_ channel currents under both normoxic and H-R conditions (**b**). Pyr3 exhibited less inhibition on both components of IK_Ca_ and SK_Ca_ induced by BK when compared with the cells without H-R exposure (**c**). *p < 0.05, **p < 0.01, one-way ANOVA and Scheffe post-hoc test (**a** and **b**) and unpaired *t* test (**c**). n = 5 in each group. N indicates the number of independent experiments, each obtained from cell isolates of different hearts.

H-R (60-30 min) exposure inhibited the whole-cell K^+^ current in PCAECs. In comparison to cells without H-R exposure (35.58 ± 2.74 pA/pF), the basal K^+^ current declined to 23.77 ± 1.70 pA/pF (p < 0.05), which is consistent with our previous report^16^. Bradykinin-induced K^+^ current was suppressed by H-R exposure (40.19 ± 1.61 vs. 65.73 ± 2.87 in normoxia, p < 0.01), in which bradykinin-induced IK_Ca_ current was decreased from 19.05 ± 3.03 to 10.24 ± 1.96 pA/pF (p < 0.01) and SK_Ca_ current from 10.63 ± 1.69 to 4.62 ± 0.43 pA/pF (p < 0.05). Biotinylation assay showed that H-R exposure suppresses the surface expression of IK_Ca_ channels whereas has no significant effect on the surface expression of SK_Ca_ channels in PCAECs (Supplementary Fig. S3). Similar to PCAECs subjected to normoxic condition in which K_Ca_ channel activity was demonstrated to be suppressed by TRPC3 inhibition, pretreatment of the H-R-exposed cells with the TRPC3 channel blocker Pyr3 also reduced bradykinin-induced K^+^ current (31.73 ± 1.10 pA/pF vs. 40.19 ± 1.61 pA/pF in H-R, p < 0.05) with significant suppression of both IK_Ca_ (5.19 ± 0.48 vs. 10.24 ± 1.96 pA/pF in H-R, p < 0.05) and SK_Ca_ (1.79 ± 0.36 vs. 4.62 ± 0.43 pA/pF in H-R, p < 0.05) channel currents (Fig. 3a and b). However, Pyr3 exhibited less inhibition on both IK_Ca_ and SK_Ca_ channels when compared with the cells without H-R exposure. The IK_Ca_ and SK_Ca_ currents inhibited by Pyr3 were 9.06 ± 1.81 and 5.15 ± 1.21 pA/pF respectively in normoxic cells and 5.12 ± 1.22 pA/pF (p < 0.05) and 3.14 ± 0.49 pA/pF (p < 0.05) in H-R exposed cells (Fig. 3c), suggesting that inhibition of TRPC3 by H-R contributes to H-R-induced IK_Ca_ and SK_Ca_ channel inhibition in PCAECs. For experiments involving Pyr3, Pyr3 was added to the bath solution just before the onset of hypoxia and lasted throughout the H-R exposure period. After H-R exposure, the cells were moved to patch clamp recording chamber and the recording was usually completed within 1 hour.

### Activation of TRPC3 channels protects endothelial K_Ca_ channels from H-R-induced inhibition

**Figure 4:**
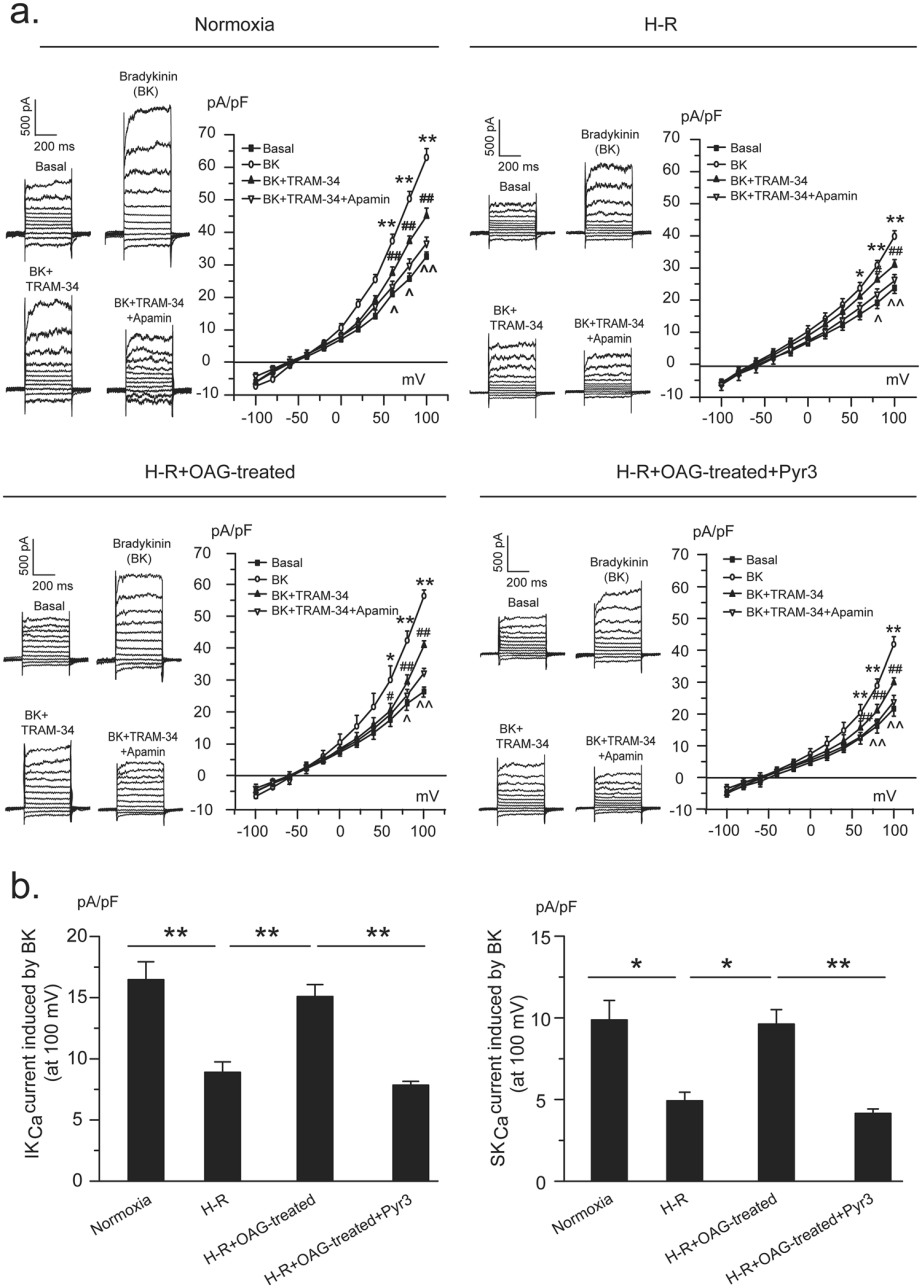
Activation of TRPC3 channels protects IK_Ca_ and SK_Ca_ channels from H-R-induced inhibition in PCAECs. (**a**) Representative traces and current-voltage relationship of whole-cell K^+^ current in PCAECs from different treatment groups before and after bradykinin (BK) stimulation with further application of the IK_Ca_ channel blocker TRAM-34 and the SK_Ca_ channel blocker apamin. Under H-R condition, PCAECs treated with OAG shows enhanced basal and BK-induced K^+^ currents compared with the cells without OAG-treatment and such enhancement is diminished by Pyr3. *p < 0.05, **p < 0.01, BK vs. basal; ^#^p < 0.05, ^##^p < 0.01, BK + TRAM-34 vs. BK; ^^^p < 0.05, ^^^^p < 0.01, BK + TRAM-34 + Apamin vs. BK + TRAM-34. (**b**) Summarized data of BK-induced IK_Ca_ and SK_Ca_ currents from 5 independent experiments, each obtained from cell isolates of different hearts. PCAECs were subjected to H-R exposure in the absence or presence of the TRPC3/6/7 activator OAG before patch-clamp recording. For cells subjected to H-R exposure and OAG treatment, the current recording was performed under the condition without (H-R + OAG-treated) or with Pyr3 application (H-R + OAG-treated + Pyr3). *p < 0.05, **p < 0.01; one-way ANOVA and Scheffe post-hoc test.

Incubation of PCAECs with OAG during H-R exposure enhanced IK_Ca_ and SK_Ca_ channel activity. OAG was added to the bath solution just before the onset of hypoxia and the subsequent current recording was performed by moving the cells to patch clamp recording chamber after H-R exposure. Compared with the H-R-exposed cells, cells exposed to H-R and co-treated with OAG exhibited significantly larger current density of IK_Ca_ (15.08 ± 0.99 vs. 8.89 ± 0.86 pA/pF in H-R, p < 0.01) and SK_Ca_ (9.62 ± 0.89 vs. 4.92 ± 0.53 pA/pF in H-R, p < 0.05) upon bradykinin stimulation, leading to increased whole-cell K^+^ current (56.67 ± 1.79 vs. 39.79 ± 0.82 pA/pF in H-R, p < 0.01). Further experiments revealed that TRPC3 channel activation mediates OAG-induced protection against H-R on K_Ca_ channel activity, which is suggested by the findings of loss of enhancement of bradykinin-induced K^+^ (38.93 ± 1.22 pA/pF, p < 0.01) and IK_Ca_ (7.94 ± 0.32 pA/pF, p < 0.01) and SK_Ca_ (4.15 ± 0.27 pA/pF, p < 0.01) channel currents in OAG-incubated cells in which current recording was performed in the presence of Pyr3 (Fig. 4a and b). Under H-R condition, activation of TRPC3 also preserves the constitutive activity of IK_Ca_ and SK_Ca_ channels, suggested by the finding of an increase of basal K^+^ currents in OAG-treated cells (28.90 ± 1.42 vs. 23.59 ± 1.75 pA/pF in H-R, p < 0.05) that is diminished by Pyr3 (22.03 ± 2.32 pA/pF) (Fig. 4a).

### Activation of TRPC3 channels preserves vasodilatory function of endothelial K_Ca_ channels under H-R condition

**Figure 5:**
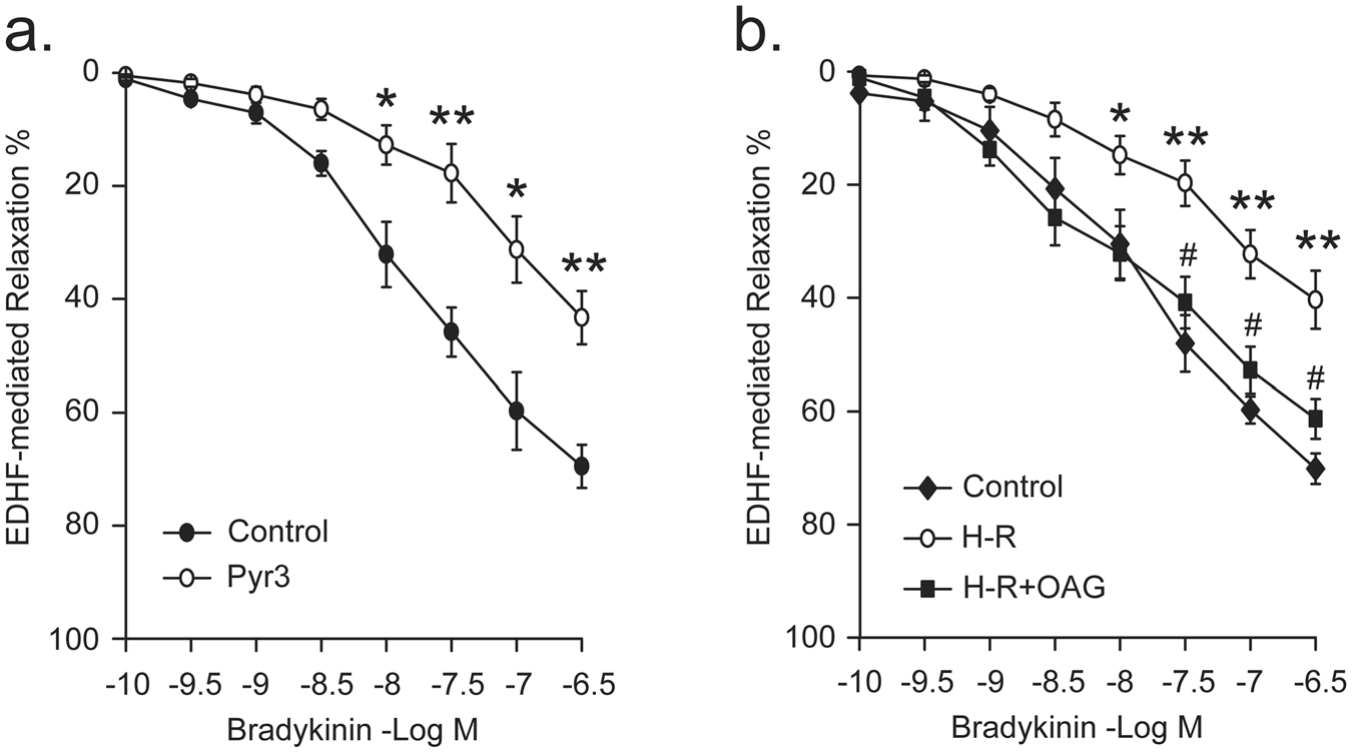
TRPC3 channels play an important role in EDHF-mediated relaxation in porcine small coronary arteries (**a**, n = 8 in each group). H-R exposure impairs the EDHF-mediated vasorelaxation and activation of TRPC3 preserves the EDHF-type response under H-R condition (**b**, n = 8 in each group). The EDHF-response was induced by bradykinin in the presence of indomethacin, N^G^-nitro-L-arginine, and oxyhemoglobin. *p < 0.05, **p < 0.01 vs. control; ^#^p < 0.05, ^##^p < 0.01 vs. H-R, unpaired *t* test (**a**), one-way ANOVA and Scheffe post-hoc test (**b**). Pyr3: TRPC3 selective blocker. OAG: TRPC3/6/7 activator.

Porcine small coronary arteries pretreated with Pyr3 showed attenuated EDHF-type relaxation to bradykinin (Rmax: 43.3 ± 4.7% vs. 69.5 ± 3.8%, p < 0.01) (Fig. 5a). Exposure to H-R compromised bradykinin-induced EDHF-mediated relaxation (Rmax: 40.4 ± 5.1% vs. 70.1 ± 2.7% in normoxic control, p < 0.01). Treatment with OAG throughout the H-R exposure period prevented the inhibition of EDHF-mediated relaxant response (61.3 ± 3.5%, p < 0.05 vs. H-R), indicating that activation of TRPC3 channels preserves K_Ca_ channel-related endothelial function under H-R condition (Fig. 5b). No differences were observed among different treatment groups with regard to resting force and the U46619-induced pre-contraction (Supplementary Table S1).

## Discussion

The present study demonstrated that in porcine coronary arteries (1) TRPC3 channels play a fundamental role in the regulation of K_Ca_ channel activity in endothelial cells; (2) the functional association between TRPC3 and K_Ca_ channels does not involve physical interaction; (3) reduction of membrane TRPC3 contributes to H-R-induced suppression of IK_Ca_ and SK_Ca_ channel activity; (4) activation of TRPC3 protects IK_Ca_ and SK_Ca_ channels from H-R-induced inhibition thereby preserving EDHF-type vasorelaxation.

Previous investigations have shown the significance of TRPC3 channels in arterial tone and blood flow regulation in both animal and human vasculatures^8, 9, 20, 21^. In our prior studies in porcine coronary arteries, we reported that TRPC3 channels regulate vascular tone through promoting NO production^8^ and EDHF-type relaxation^19^. The present study further demonstrated that TRPC3 channels participate in EDHF-type relaxation through mediating activation of endothelial IK_Ca_ and SK_Ca_ channels, which is in agreement with the findings in rodent vascular beds^17, 18^. By using sharp electrode to measure membrane potential, Senadheera and colleagues observed that in the presence of cyclooxygenase and eNOS inhibitors, inhibition of TRPC3 channels with Pyr3 significantly reduced the amplitude of ACh-induced hyperpolarization in rat mesenteric artery endothelial cells, which was the first published electrophysiological evidence suggesting the functional role of TRPC3 in K_Ca_-dependent EDHF mechanism. A subsequent study by the same group on mouse cerebral arteries yielded similar results^18^ and further linked TRPC3 channel activation and SK_Ca_ channel activation in the EDHF-response with support of channel current recording data. In the present study, we observed that in PCAECs, pharmacological inhibition of TRPC3 channels and gene silencing of TRPC3 significantly suppressed TRAM-34-sensitive and apamin-sensitive whole-cell K^+^ currents in response to bradykinin, suggesting the requirement of TRPC3 channels in the activation of both IK_Ca_ and SK_Ca_ channels in porcine coronary endothelium.

Given the knowledge that TRP channels may associate with other proteins to form a signaling complex, for example, colocalization of TRPC1^22^ or TRPC3/6^23^ with BK_Ca_ was found to underlie the role of these TRP members in the activation of BK_Ca_ channels. We investigated whether the functional link between TRPC3 and IK_Ca/_SK_Ca_ channels involves direct physical association of the channel proteins. Our co-immunoprecipitation experiments showed that in PCAECs, TRPC3 protein neither binds with K_Ca_3.1 nor K_Ca_2.3 protein, suggesting that TRPC3 does not physically interact with IK_Ca/_SK_Ca_ channels to regulate the K_Ca_ channel activity in porcine coronary endothelium. As to the study of SK_Ca_ channel proteins, among the three subtypes SK1 (K_Ca_2.1), SK2 (K_Ca_2.2) and SK3 (K_Ca_2.3), we focused on SK3 (K_Ca_2.3), which is because (1) previous investigation demonstrated that no mRNA encoding SK1 is detected in porcine coronary endothelium and the apamin-sensitive component of the EDHF-response is mediated by SK3 while not by SK2^24^, and (2) our latest study added a further piece of evidence concerning the significance of K_Ca_2.3 subtype in the regulation of endothelial function in this vasculature^25^.

It has been reported that in vascular endothelial cells IK_Ca_ channels predominantly reside in myoendothelial projections and SK_Ca_ channels preferentially locate at the sites of homocellular endothelial gap junctions and caveolin-rich domains^26^. In rat mesenteric arteries, based on the findings of strong expression of TRPC3 in myoendothelial contact regions, Senadheera and colleagues proposed that a close proximity of TRPC3 and IK_Ca_ channels facilitates IK_Ca_ activation via TRPC3-dependent Ca^2+^ signaling that may involve Ca^2+^ influx and/or refilling of the IP_3_R-mediated endoplasmic reticulum Ca^2+^ stores^17^. The exact localizations of TRPC3 and K_Ca_ channels were not characterized in the present study, however, with the findings of no binding of TRPC3 with K_Ca_ proteins whereas significant role of TRPC3 in K_Ca_ channel activity, the present study favors the theory of TRPC3-mediated Ca^2+^ signals for K_Ca_ channel activation in PCAECs. Further studies of how Ca^2+^ signals bridge TRPC3 and K_Ca_, e.g., initiation and location of the signal, and/or cascades involved in Ca^2+^ signaling, need to be undertaken in order to fully understand the functional role of TRPC3 in K_Ca_ channel activation.

The most important and novel finding of the present study is that inhibition of TRPC3 channels contributes to H-R-induced suppression of channel activity of IK_Ca_ and SK_Ca_. We previously reported that H-R suppresses K_Ca_ and TRPC3 channel activity in porcine coronary arterial endothelial cells^8, 16^. In the present study, given our demonstration that TRPC3 is integral to endothelial IK_Ca_ and SK_Ca_ channel activation in porcine coronary artery, we further explored the relationship between TRPC3 channel inhibition and K_Ca_ channel inhibition and its significance in endothelial dysfunction related to I-R injury. Patch-clamp data showed that in both control and H-R-exposed PCAECs, TRPC3 channel blocker Pyr3 inhibits bradykinin-induced IK_Ca_ and SK_Ca_ channel currents, however, the extent of inhibition is significantly less in H-R-exposed cells in comparison with cells without H-R exposure (Fig. 3). Further recordings showed that activation of TRPC3 channels effectively prevents H-R-induced suppression of endothelial K_Ca_ channel activity (Fig. 4). Both basal K^+^ currents and IK_Ca_ and SK_Ca_ currents in response to bradykinin were enhanced in cells exposed to H-R and co-treated with OAG, in which blocking TRPC3 channels with Pyr3 diminished the protective effect conferred by OAG. In our previous study, we demonstrated that in our preparation of PCAECs, the basal K^+^ current is predominantly composed of IK_Ca_ and SK_Ca_ currents^16^. Taken together, these results suggest that loss of TRPC3 channel activity contributes to H-R-induced IK_Ca_ and SK_Ca_ channel inhibition in coronary endothelium. In conjunction with the finding of decreased cell surface expression of IK_Ca_ (K_Ca_ 3.1) and insignificant change in cell surface expression of SK_Ca_ (K_Ca_2.3) following H-R exposure, our results suggest that the inhibition of IK_Ca_ current induced by H-R is a combined effect of less IK_Ca_ in the membrane and less Ca^2+^ (due to less TRPC3) whereas H-R-induced inhibition of SK_Ca_ current is primarily due to the less Ca^2+^ for activation. The significance of TRPC3 inhibition in K_Ca_ inhibition therefore provided a mechanistic explanation for the beneficial effect of activation of TRPC3 channels on EDHF function in H-R-exposed coronary arteries (Fig. 5).

Recent evidence suggests that trafficking of TRPC3 to the plasma membrane facilitates SK_Ca_ channel activation in mouse cerebral endothelium^18^. In a prior study, we demonstrated that in H-R-exposed PCAECs, decreases of TRPC3 channel currents and TRPC3-mediated Ca^2+^ entry are attributable to the inhibition of TRPC3 trafficking that results in reduction of TRPC3 channels presented at the endothelial cell surface^8^. In light of these findings, we therefore propose that inhibition of TRPC3 trafficking is the likely mechanism underlying the pathological significance of TRPC3 in endothelial K_Ca_ channel dysfunction associated with H-R injury.

This study has several limitations that must be acknowledged. The negative results of our co-immunoprecipitation experiments suggest that TRPC3 and K_Ca_ channels are not physically associated with each other in PCAECs. In the present study, the protein-protein interactions of TRPC3 and either IK_Ca_ and SK_Ca_ were only studied in endothelial cells under unstimulated condition and no further efforts were made to explore whether these may change when cells are upon stimulation, e.g., by agonists such as bradykinin, or by pathological factors such as I-R/H-R. Considering the fact that internal and external stimuli may dynamically regulate ion channels through mechanisms including, but not limited to, forward trafficking and endocytosis of channels, further studies of protein-protein interaction of TRPC3 and K_Ca_ in stimulated condition is therefore necessary to derive a complete and dynamic understanding of the relationship of these two channels in the control of coronary arterial tone. In addition, although we demonstrated that TRPC3 physically interacts with neither IK_Ca_ nor SK_Ca_ channels in PCAECs, current results could not answer the question whether TRPC3 and K_Ca_ channels are colocalized or located in close proximity in these cells. Further studies with double immunofluorescence staining and fluorescence resonance energy transfer measurement shall reveal the spatial proximity between these channels thereby deepen our understanding of how TRPC3 functions to regulate K_Ca_ channel activity under physiological condition and how H-R disrupts the functional interaction between TRPC3 and K_Ca_.

In conclusion, the present study demonstrated that TRPC3 channels play an important role in IK_Ca_ and SK_Ca_ channel activation in coronary arterial endothelial cells. The functional association between TRPC3 and K_Ca_ channels does not involve physical interaction. Inhibition of TRPC3 channels contributes to H-R-induced suppression of IK_Ca_ and SK_Ca_ channel activity, which serves as a mechanism underlying endothelial dysfunction under H-R condition and renders TRPC3 channels a potential target for endothelial protection in ischemic status.

## Materials and Methods

Fresh hearts of young adult pigs (~35 kg) were collected from a local slaughterhouse. Once excised, the hearts were placed in a container filled with cold (4 °C), pre-oxygenated (95%O_2_/5%CO_2_) Krebs solution and immediately transferred to the laboratory for dissection of small coronary arteries as in our previous studies^8, 16, 19^. All experiments were in accordance with institutional guidelines and approved by the Animal Experimentation Ethics Committee, and Safety Office of the Chinese University of Hong Kong (Ref. No. 14/010/GRF).

### Isolation and primary culture of porcine coronary arterial endothelial cells (PCAECs)

PCAECs were isolated by enzymatic digestion from porcine small coronary arteries and cultured as previously described^8, 16, 19^. Briefly, small porcine coronary arteries taken from the branches of left anterior descending artery (LAD) were dissected into tiny strips and treated with 0.2% collagenase (type I, Sigma) at 37 °C for 15 min. The suspension was centrifuged at 1600 rpm for 5 min and cells were resuspended in 5 mL culture medium containing 90% RPMI and 10% FBS with 100 U/mL penicillin and 100 μg/mL streptomycin. After 2-hour incubation at 37 °C, the medium was replaced once to remove unattached cells. Attached endothelial cells were cultured in medium containing antibiotics in a humidified incubator with 5% CO_2_ at 37 °C for ~6 days. For maintaining electrophysiological properties of isolated coronary endothelial cells, only primary cells were used for experiments. The endothelial nature of the cultured cells was identified by immunostaining for von Willebrand Factor (vWF) as previously described^8^. Briefly, cultured cells seeded on glass coverslips were fixed with 4% formaldehyde for 15 min at room temperature and blocked in PBS containing 2% BSA for 60 min, then incubated overnight at 4 °C with rabbit anti-vWF antibody (1:200), followed by 1 hour incubation with Alexa Fluor® 488-conjugated goat anti-rabbit IgG (Abcam, 1:500) at room temperature in the dark. Immunostaining for alpha-smooth muscle actin (α-SMA) was used to evaluate the contamination by smooth muscle cells, which was performed by incubation of the cells with anti-α-SMA antibody (1:200) followed by Alexa Fluor® 555-conjugated donkey anti-rabbit antibody (Abcam, 1:500). The coverslip was placed on a microscope slide and covered with a drop of ProLong Gold antifade reagent with DAPI (Invitrogen). After rinsing with PBS, the cells were observed and photographed using a fluorescent microscope (Leica DM 6000 FS, Germany).

### Whole-cell recording using the perforated patch-clamp technique for IK_Ca_, SK_Ca_, and TRPC3 channel currents in PCAECs

Whole-cell K^+^ currents of PCAECs were recorded by using the whole-cell perforated patch-clamp technique (EPC10, HEKA, Lambrecht, Germany) at room temperature (20–24 °C) with further differentiation of the IK_Ca_ and SK_Ca_ components^16, 25^. Briefly, patch pipettes with resistance of 3–5 MΩ were filled with a solution containing (mmol/L): NaCl 20, KCl 40, K-aspartate 80, MgCl_2_ 1, Mg-ATP 3, EGTA 10, and K-HEPES 5 (pH adjusted to 7.2 using KOH). The pipette solution contains amphotericin B (500 μg/mL) as the perforating agent. The bath solution contains (mmol/L): NaCl 140, KCl 5.4, MgCl_2_ 1, CaCl_2_ 1.8 and Na-HEPES 10 (pH 7.4 adjusted with NaOH). Whole-cell currents were evoked by 500-ms voltage steps from −100 mV to +100 mV in 20 mV increments from a holding potential of −60 mV. K^+^ currents induced by bradykinin (100 nmol/L) were recorded with further application of the specific IK_Ca_ channel blocker TRAM-34 (1 μmol/L) followed by SK_Ca_ blocker apamin (100 nmol/L)^24, 25, 27^. Currents were recorded after achievement of the full perforation of the membrane patch that usually took 3–5 min, as judged from the development of repeatable currents in response to step depolarizations. Liquid junction potentials, series resistance, and cell capacitance were compensated. For recording of TRPC3 channel currents, patch pipettes were filled with a solution containing (mmol/L): Cs-methanesulphonate 120, CsCl 20, HEPES 10, MgCl_2_ 1, and EGTA 1 (pH 7.2 with KOH). The bath solution contains (mmol/L): NaCl 137, KCl 5.4, HEPES 10, glucose 10, MgCl_2_ 1, and CaCl_2_ 2 (pH 7.4 with NaOH). Membrane currents were recorded in whole-cell mode using a voltage-clamp protocol (voltage ramps from −100 to +100 mV, 0.6 V/s, 0.2 Hz, holding potential −70 mV). Current in response to the TRPC3/6/7 activator 1-oleoyl-2-acetyl-sn-glycerol (OAG) (100 µmol/L) were recorded with further application of the TRPC3 selective blocker Pyr3 (10 µmol/L)^8^ to differentiate the TRPC3 component. Data were analyzed with PulseFit software (HEKA). The current was normalized by cell capacitance into current densities (pA/pF). Only recordings with seal resistance >500 MΩ, and series resistance <10 MΩ were included in the study and experiments were only analyzed if changes in series resistance were no more than 2 MΩ in the entire recording period.

### siRNA knockdown of endogenous TRPC3 channels

Primary cultured PCAECs were transfected with the TRPC3-specific siRNA (sense: 5′GCUCGAGGAUCAAUGCCUATT3′, antisense: 5′UAGGCAUUGAUCCUCGAGCTT3′) by using LipofectamineTM 2000 (Invitrogen), according to manufacturer’s instructions. Knockdown efficiency of TRPC3 was determined at both mRNA level by RT-PCR and protein level by western blotting.

### RT-PCR analysis of mRNA expression of TRPC3

Total RNA from PCAECs was extracted using RNAiso reagent (Takara) and mRNA was converted to cDNA using PrimeScriptTM RT Master Mix (Takara), according to manufacturer’s instructions. PCR amplification was performed using GoTaq® G2 Flexi DNA Polymerase (Promega). For TRPC3 amplification, PCR parameters were as follows: 95 °C activation (30 s) followed by 95 °C dissociation (30 s), 46.6 °C anneal (30 s), and 72 °C elongation (1 min) for 30 cycles. Primers used for TRPC3 amplification were: forward: 5′GCAACAAAGGCACAGCAGTA3′, reverse: 5′TTGAGCACAACGGAAGTCAC3′. As the internal loading control, GAPDH was amplified for 30 cycles with the forward primer 5′GGTCGGAGTGAACGGATTT3′ and the reverse primer 5′ATTTGATGTTGGCGGGAT3′ under the following condition: activation (95 °C, 30 s) - dissociation (94 °C, 30 s) - anneal (43.3 °C, 30 s) - elongation (72 °C, 1 min). Products were identified by electrophoresis and digitally imaged for analysis (Geliance 600 Imaging System, PerKinElmer, UK).

### Western blot analysis of protein expression of TRPC3

Whole cell protein was extracted from primary cultured PCAECs with ice-cold RIPA lysis buffer, composed of 50 mmol/L Tris-HCl (pH 7.5), 150 mmol/L NaCl, 3.75 mmol/L KCl, 1% NP-40, 0.1% sodium dodecyl sulfate, and 0.5% sodium deoxycholate. Protease (1 tablet in 50 ml) and phosphatase (1 tablet in 10 ml) inhibitor cocktail tablets were freshly added to the buffer before use (Roche Diagnostics). The protein extraction was centrifuged at 4 °C for 20 min at 12000 rpm and the supernatant was then mixed with loading buffer, heated up to 100 °C for 10 min, then fractionated by denaturing 8% sodium-dodecyl-sulfate polyacrylamide-gel electrophoresis (25 µg per lane) for 90 min at 120 V and electro-transferred to polyvinylidene difluoride membrane (Thermo scientific) for 90 min at 100 V. The membrane was blocked with 5% BSA/TBST for 1 hour at room temperature and incubated with the primary antibody against TRPC3 (1:1000 dilution, Abcam) or β-tubulin (1:2500, Abcam) in 5% BSA/TBST overnight at 4 °C. The membrane was then washed in TBST followed by incubation with secondary IRDye800®-infrared fluorescent dye-conjugated goat anti-rabbit antibody (1:10000, Rockland) in TBST for 1 hour at room temperature. Imaging was performed by using Odyssey gel imaging scanner (Li-Cor Biosciences) at a wavelength of 800 nm and band intensities were analyzed by Quantity One imaging software (Version 4.6.6, Bio-Rad). β-tubulin served as internal loading control.

### Co-immunoprecipitation

Experiments were performed using methods described elsewhere^28^ with slight modifications. Proteins were extracted from PCAECs using a detergent extraction buffer containing 1% (vol/vol) Nonidet P-40, 150 mmol/L NaCl, and 20 mmol/L Tris-HCl, pH 8.0, with the addition of protease and phosphatase inhibitor cocktail (Roche Diagnostics). Extracted protein (1000 μg) was incubated overnight with 10 μg of antibody against TRPC3 (Alomone Labs), K_Ca_2.3 (Alomone Labs), or K_Ca_3.1 (Alomone Labs) at 4 °C on a rocking platform. Protein A agarose was added and incubated at 4 °C for another 4 hours. Immunoprecipitates were then washed twice with saline and resolved on 8% sodium dodecyl sulphate-polyacrylamide electrophoresis gel. For immunoblots, the polyvinylidene difluoride membrane carrying the transferred proteins was incubated at 4 °C overnight with designated primary antibodies diluted (1:200) in TBST buffer containing 0.1% Tween 20 and 5% BSA. Immunodetection was accomplished by 1-hour incubation with secondary IRDye800®-infrared fluorescent dye-conjugated goat anti-rabbit antibody (1:10000, Rockland) in TBST at room temperature. Imaging was performed at a wavelength of 800 nm with using Odyssey gel imaging scanner (Li-Cor Biosciences). To complete the protein-protein interaction analysis of TRPC3 and K_Ca_ channels, immunoprecipitate obtained with anti-TRPC3 antibody was subjected to immunoblot analysis using anti-K_Ca_3.1 or anti-K_Ca_2.3 antibodies. The immunoprecipitation and immunoblot experiments were then performed in reversed order, i.e., immunoprecipitation with anti-K_Ca_3.1 or anti-K_Ca_2.3 antibody, followed by immunoblotting with anti-TRPC3 antibody.

### Isometric force study

Porcine small coronary arteries with the diameter of 300~500 µm were dissected from the branches of LAD and cut into cylindrical rings with 2-mm in length. The rings were mounted, normalized, and equilibrated in a four-channel Mulvany myograph (Model 610 M, J.P. Trading, Aarhus, Denmark) as previously described^8, 16, 19, 25^. The IK_Ca_ and SK_Ca_ channel-dependent, EDHF-mediated relaxation was studied in U_46619_-preconstricted rings upon bradykinin (−10~−6.5 LogM) stimulation in the presence of cyclooxygenase inhibitor indomethacin (Indo, 7 μmol/L), NOS inhibitor N^G^-nitro-L-arginine (_L_-NNA, 300 μmol/L), and NO scavenger oxyhemoglobin (HbO, 20 μmol/L)^29, 30^.

### Induction of H-R

As described in our previous studies^8, 16, 31^, small coronary arteries or primary cultured PCAECs (seeded on glass coverslips) were placed in a plastic cover-sealed chamber filled with bath solution that was continuously bubbled with 95%N_2_-5%CO_2_ at 37.0 ± 0.1 °C to induce hypoxia exposure (PO_2_ < 5 mmHg, monitored by Oxygen Meter Model 781, Strathkelvin Instrument, Glasgow, Scotland, UK) for 60 min, followed by 30 min of reoxygenation before isometric force study and voltage-clamp recording in relevant protocols. For studies using coronary arteries, the bath solution was Krebs solution (pH 7.4) that contains (in mmol/L): Na^+^ 143.4, K^+^ 5.9, Ca^2+^ 2.5, Mg^2+^ 1.2, Cl^−^ 128.7, HCO_3_^−^ 25, SO_4_^2−^ 1.2, H_2_PO_4−_ 1.2, and glucose 11.1. While in studies with PCAECs, the bath solution was RPMI-1640 medium.

### Data analysis

Data were presented as mean ± s.e.m. Statistical analyses were performed using unpaired *t* test or one-way ANOVA followed by Scheffe test (SPSS, version 20) when appropriate. Differences were considered significant when p < 0.05.

## Electronic supplementary material

Supplementary Information
