# Supplementary material for: Impairment of Coronary Endothelial Function by Hypoxia-Reoxygenation Involves TRPC3 Inhibition-mediated KCa Channel Dysfunction: Implication in Ischemia-Reperfusion Injury
Source: Sci Rep. 2017 Jul 19;7:5895. doi: 10.1038/s41598-017-06247-3 (PMC5517640; doi:10.1038/s41598-017-06247-3)
Supplement: Supplementary file 1 — Supplementary Information [file 41598_2017_6247_MOESM1_ESM.doc]

**Supplementary Information**

**Impairment of Coronary Endothelial Function by Hypoxia-Reoxygenation Involves TRPC3 Inhibition-mediated K_Ca_ Channel Dysfunction: Implication in Ischemia-Reperfusion Injury**

Xiang-Chong Wang, *M.Phil* ^1^; Wen-Tao Sun, *M.Phil* ^1^; Jie Fu, *M.Phil* ^1^; Jun-Hao Huang, *PhD* ^2,3^; Cheuk-Man Yu, *MD*^1^; Malcolm John Underwood, *MD*^2^; Guo-Wei He, *MD, PhD* ^4^;

Qin Yang, *MD, PhD* ^1,4^*

^1^Division of Cardiology, Department of Medicine and Therapeutics, Institute of Vascular Medicine, Li Ka Shing Institute of Health Sciences, Institute of Innovative Medicine, The Chinese University of Hong Kong

^2^Division of Cardiothoracic Surgery, Department of Surgery, The Chinese University of Hong Kong, Hong Kong

^3^Guangzhou Sport University, GuangZhou, China

^4^TEDA International Cardiovascular Hospital, Chinese Academy of Medical Sciences, Tianjin, China

*Correspondence to

**Professor Qin Yang**, MD, PhD

Division of Cardiology

Department of Medicine and Therapeutics

The Chinese University of Hong Kong

Prince of Wales Hospital, Shatin, N.T., Hong Kong

E-mail: yangqs@cuhk.edu.hk

**Method**

**Biotinylation assay of cell surface expression of IK_Ca_ and SK_Ca_ channels**

Cell surface protein isolation of PCAECs was performed using the Pierce cell surface protein isolation kit (Thermo Scientific) as in our previous studies ^8,25^. Briefly, PCAECs with or without H-R exposure were washed with ice-cold PBS and labelled with EZ-Link Sulfo-NHS-SS-Biotin for 30 min at 4°C on rocking platform. After adding quenching solution, the cells were scraped off and lysed in lysis buffer containing protease and phosphatase inhibitors cocktail for 30 min on ice followed by centrifuge at 10000×g at 4°C for 2 min. Biotin-labeled cell surface proteins were collected by NeutrAvidin Agarose in a spin column and eluted by sample buffer containing 62.5 mmol/L Tris-Hcl (pH 6.8), 1% SDS, 10% glycerol and 50 mmol/L dithiothreitol into a collection tube. The eluent was heated for 5 min at 95°C and bromophenol blue was added. The concentration of the isolated plasma membrane protein was determined and protein levels of K_Ca_3.1and K_Ca_2.3 were detected by Western blot.

**Supplementary Table S1.** Resting force and precontraction induced by U46619 in porcine small coronary arteries subjected to different treatments.

| Group | Resting force  (mN) | U46619-induced precontraction  (mN) |
| --- | --- | --- |
| *In Fig.5a* |  |  |
| Control | 3.2±0.4 | 9.5±1.0 |
| Pyr3 | 2.8±0.3 | 10.2±2.3 |
| *In Fig.5b* |  |  |
| Control | 2.9±0.2 | 8.3±0.7 |
| H-R | 2.7±0.2 | 8.8±1.2 |
| H-R+OAG | 3.0±0.3 | 9.4±1.6 |

Data are shown as mean±SEM. n=8 in each group.

**Supplementary Figure S1.**

**a.** **b.**


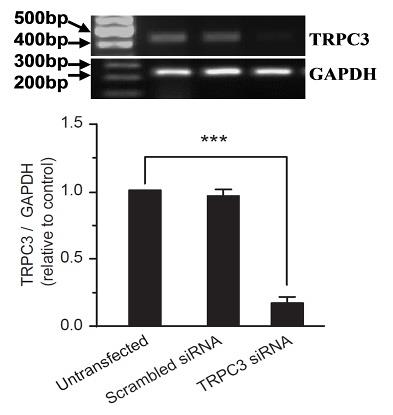

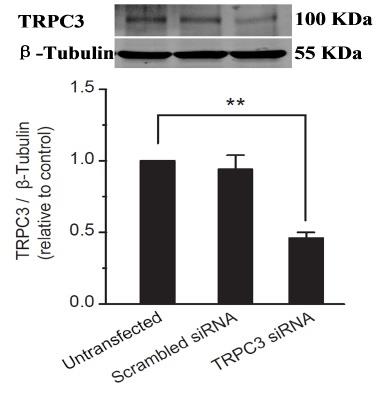


**c.** **d.**


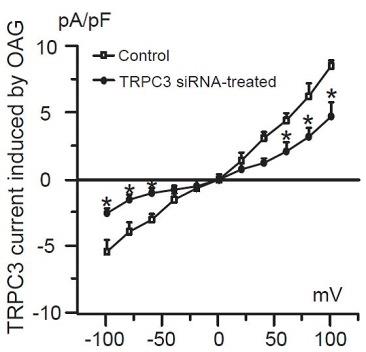


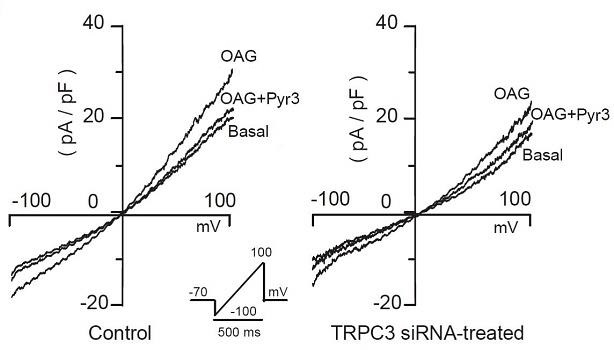


**Suppl Fig.S1.** TRPC3 channel blockade and knockdown of TRPC3 significantly suppress IK_Ca_ and SK_Ca_ channel currents in PCAECs. Efficient knockdown of TRPC3 with siRNA was evidenced by dramatic decreases in both mRNA and protein expressions of TRPC3 **(a & b)**, and significant suppression of TRPC3 channel current **(c & d)**. **(c)** The current-voltage relationship of OAG (TRPC3/6/7 activator)-induced membrane currents before and after application of the selective TRPC3 channel blocker Pyr3; **(d)** Summarized data of OAG-induced TRPC3 channel current from 5 independent experiments, each obtained from cell isolates of different hearts.**^*^**p<0.05, **^**^**p<0.01, **^***^**p<0.001; one-way ANOVA and Scheffe post-hoc test (a & b) and unpaired *t* test (d).

**Supplementary Figure S2.**

**
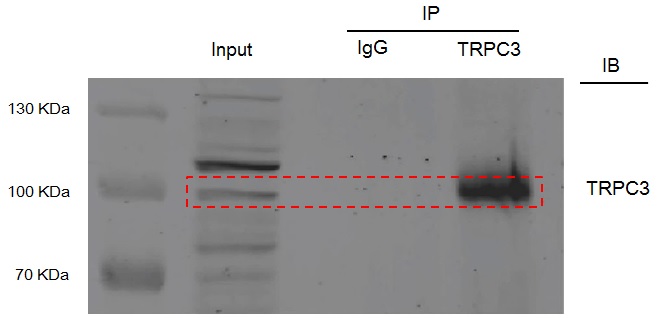
**


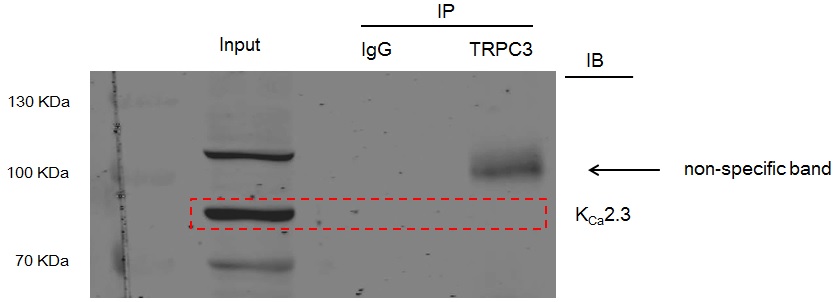


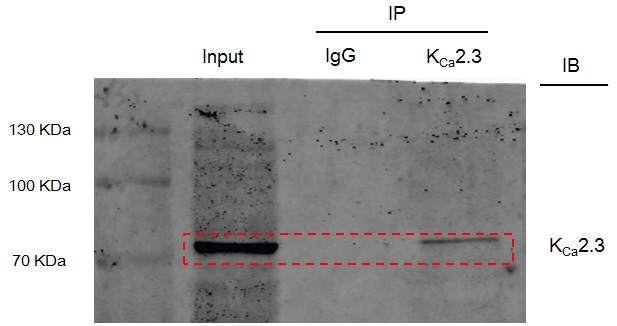


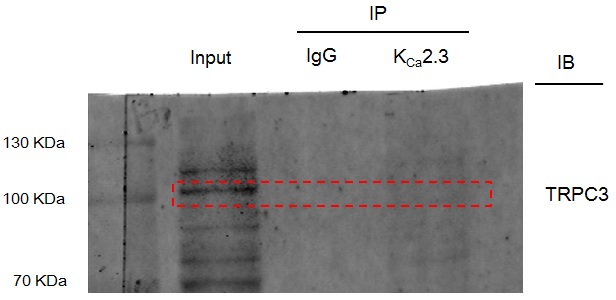


**Suppl Fig.S2.** Full-length western blot images of the cropped blots shown in Figure 2. The hatched red line indicates the band corresponding to the target protein.

**Supplementary Figure S3.**

**
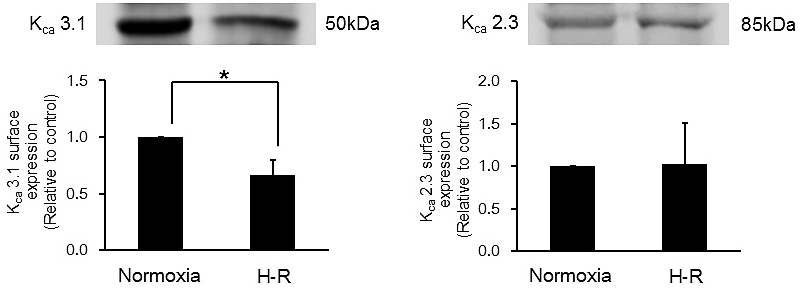
**

**Suppl Fig. S3.** H-R exposure suppresses the surface expression of IK_Ca_ channels whereas has no significant effect on the surface expression of SK_Ca_ channels in PCAECs. Surface protein expression was determined by biotinylation assay. *p<0.05, n=4, unpaired t-test.

**Supplementary Figure S4.**

**
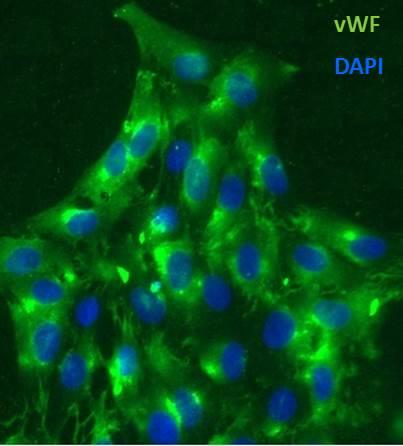
**

**Suppl Fig.S4.** Immunofluorescent staining of the cultured cells with the antibody of Von Willebrand Factor (VWF) demonstrates the endothelial nature of the cells. DAPI was applied to stain the nucleus.
